# Long-term changes in the refractive effect of a toric intraocular lens on astigmatism correction

**DOI:** 10.1007/s00417-021-05406-7

**Published:** 2021-09-08

**Authors:** Ken Hayashi, Motoaki Yoshida, Shunsuke Hayashi, Akira Hirata

**Affiliations:** 1 Hayashi Eye Hospital, 4-7-13 Hakataekimae, Hakata-Ku, Fukuoka, 812-0011 Japan; 2 Department of Ophthalmology, National Hospital Organization of Saitama Hospital, Wako, Japan; 3 Department of Ophthalmology, Faculty of Medicine, Keio University, Tokyo, Japan

**Keywords:** Cataract surgery, Long-term astigmatic changes with age, Toric intraocular lens, Against-the-rule astigmatism, With-the-rule astigmatism

## Abstract

**Purpose:**

To examine the long-term changes in the astigmatism-correcting effect of a toric intraocular lens (IOL) after stabilization of surgically induced astigmatic changes due to cataract surgery.

**Methods:**

Unilateral eyes of 120 patients that received a toric IOL for against-the-rule (ATR) or with-the-rule (WTR) astigmatism were enrolled. Manifest refractive and anterior corneal astigmatism, and ocular residual astigmatism which is mainly derived from internal optics were examined preoperatively, at approximately 2 months postoperatively (baseline) and at 5 ~ 10 years postbaseline. The astigmatism was decomposed to vertical/horizontal (Rx) and oblique components (Ry), which was compared between baseline and 5 ~ 10 years postbaseline.

**Results:**

In the eyes having ATR astigmatism, the mean Rx and Ry of the manifest refractive and corneal astigmatism significantly changed toward ATR astigmatism between the baseline and 5 ~ 10 years postbaseline (*p* ≤ 0.0304), but those of ocular residual astigmatism did not change significantly between the 2 time points. In the eyes having WTR astigmatism, the Rx and Ry of refractive, corneal, and ocular residual astigmatism did not change significantly between the 2 time points. Double-angle plots revealed an ATR shift in refractive and corneal astigmatism and no marked change in the ocular residual astigmatism in the eyes with ATR astigmatism, and there is no change in this astigmatism in the eyes with WTR astigmatism.

**Conclusion:**

The long-term changes with age in the effect of a toric IOL significantly deteriorated due to an ATR shift of corneal astigmatism in the eyes having ATR astigmatism, while it was maintained in eyes having WTR astigmatism, suggesting that ATR astigmatism should be overcorrected.

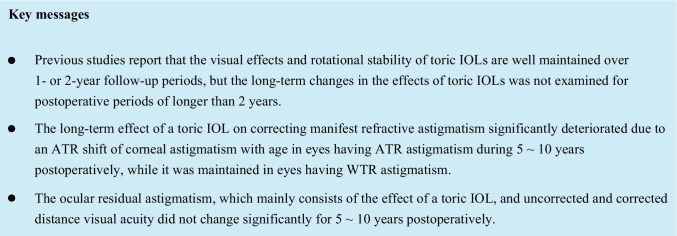

**Supplementary Information:**

The online version contains supplementary material available at 10.1007/s00417-021-05406-7.

## Introduction

Toric intraocular lenses (IOLs) effectively correct preexisting corneal astigmatism when implanted during cataract surgery and substantially improve uncorrected visual acuity [1–5]. Many novel devices have been developed for precisely aligning the meridian of toric IOLs, including image-guided system and intraoperative aberrometers, resulting in improvements in short-term visual outcomes [6–9]. Additionally, multifocal IOLs with a toric component can enhance the multifocal effect in eyes with excessive preexisting corneal astigmatism [10–13].

The long-term changes in the visual effects of toric IOLs on astigmatic correction remain unclear. Some studies report that the visual effects and rotational stability of toric IOLs are well maintained over 1- or 2-year follow-up times [14–18]. For example, Miyake et al. [16] reported excellent visual outcomes and rotational stability for up to 2 years after surgery. The long-term changes in the effects of toric IOLs for astigmatism correction after stabilization of surgically induced astigmatic change, however, have not been evaluated for longer postoperative intervals.

The present study examined the changes with age in the astigmatism-correcting effects of toric IOLs for 5 ~ 10 years after stabilization of surgically induced astigmatic changes. Because astigmatism in the posterior cornea is an against-the-rule (ATR) astigmatism in most eyes and has a non-negligible effect in determining the IOL power [19, 20], the refractive effects may differ between eyes having ATR astigmatism and eyes having with-the-rule (WTR) astigmatism when the toric IOL cylindrical power and model are selected on the basis of data from only the anterior cornea [21–23]. Accordingly, in the present study, the long-term changes in the effects of a toric IOL were separately evaluated in eyes having preoperative ATR or WTR astigmatism.

## Patients and methods

### Study design

This study was a retrospective observational study conducted at the Hayashi Eye Hospital, Fukuoka, Japan, between February 26 and October 14, 2020. The study protocol was approved by the Institutional Review Board of the Hayashi Eye Hospital on February 25, 2020. The study protocol adhered to the tenets of the Declaration of Helsinki.

### Participants

The medical records of all patients who had undergone cataract surgery with a toric IOL implantation performed by a single surgeon (KH) between 2010 and 2015 were reviewed beginning on February 26, 2020. The eyes that were continuously followed up at the Hayashi Eye Hospital for 5 ~ 10 years after surgery were included. Inclusion criteria were (1) eyes that underwent phacoemulsification using an approximately 2.4-mm clear corneal incision; (2) eyes that received a 1-piece hydrophobic acrylic IOL with a toric component (SA60AT or SN60AT; Alcon Laboratories, Fort Worth, Texas, USA); (3) eyes that were examined at least twice using an autokerato/refractometer at approximately 2 months postoperatively, and a difference in corneal astigmatism between the 2 examinations was within 0.5 D in cylindrical power and within ± 15° in the cylindrical axis (the latter examination was defined as baseline); (4) eyes that underwent examinations using an autokerato/refractometer at 5 ~ 10 years after the baseline measurement; (5) first-operated eye when the patient underwent cataract surgery in both eyes; (6) eyes that underwent uneventful cataract surgery with no sutures; (7) eyes with no severe pathology of the cornea, optic nerve, or macula; and (8) eyes without previous history of other surgery or inflammation. Patient enrollment was continued until 120 eyes (60 eyes in each of the ATR and WTR astigmatism groups) were enrolled in the study, with the last eye enrolled on July 30, 2020.

### IOL power calculation and toric meridian determination

The spherical power of the IOL was calculated using the SRK/T formula with the optimized A-constant. For IOL power determination, the axial length was measured using the IOLMaster (Carl Zeiss Meditec GmbH, Jena, Germany). In the eyes with a dense cataract, the axial length was measured using an ultrasonic applanation biometer (Ocuscan™ Alcon Biophysic, Ferrand, France). The corneal curvature at the steepest and flattest meridians of the anterior cornea was measured by anterior segment optical coherence tomography (CASIA 1; TOMEY, Tokyo Japan), and the mean value of both meridians was used. The appropriate model and meridian of the toric IOL to achieve emmetropia was determined using an online toric IOL calculator program (available at http://www.acrysoftoriccalculator.com; Alcon). Preoperative keratometry and biometry data, incision location and size, and the surgeon’s estimated surgically induced corneal astigmatism were entered into this calculator program.

The appropriate meridian of the toric IOL was determined utilizing the CASIA 1 dataset. Prominent structures of the iris or conjunctival vessels were selected as reference points on the screen of the CASIA 1, and the meridian of the toric IOL was indicated by the clockwise or counterclockwise angle from the reference structures. The angles from the reference points were used to place the toric IOL at the appropriate meridian.

### Surgical procedures

A single surgeon (KH) performed all cataract surgeries [13]. Just before surgery, with the patient seated upright at the slit-lamp to compensate for cyclorotation, the surgeon marked the corneal limbus at 0°, 90°, 180°, and 270° using a marker after vertical alignment of the patient’s head. At the beginning of the surgery, the steepest meridian of the corneal limbus was identified and marked by a steel knife using a toric IOL marker (9-705R-1; Duckworth & Kent, Hertfordshire, UK) according to the pre-marked points at the corneal limbus and the pre-determined reference points on the iris or conjunctival structures. The surgeon made an approximately 2.4-mm clear corneal incision horizontally for eyes having ATR corneal astigmatism, and superiorly for eyes having WTR astigmatism; the horizontal incision was made temporally in the right eye and nasally in the left eye. Phacoemulsification surgery was performed according to the previously described procedure [13]. First, a continuous curvilinear capsulorrhexis measuring approximately 5.0 mm in diameter was made using a bent needle. After hydrodissection, phacoemulsification of the nucleus and aspiration of the residual cortex was conducted. Without enlarging the incision, the lens capsule was inflated with sodium hyaluronate 1% (Healon; Johnson & Johnson Vision Santa Ana, California, USA or Hyaguard; Nitten Pharmaceutical, Tokyo, Japan), after which the IOL was placed into the capsular bag. The toric IOL was rotated until the toric IOL indentations were aligned with the marked steepest meridian of corneal astigmatism. All viscoelastic materials were thoroughly aspirated, while carefully avoiding rotation of the IOL. IOL meridian alignment was confirmed after wound closure using stromal hydration. In this series, all surgeries were uneventful, and no sutures were required in any of the cases.

### Outcome measures

All patients underwent examinations preoperatively and at approximately 2 months postoperatively (baseline) and at 5 ~ 10 years after baseline. Manifest refractive spherical and cylindrical powers were measured objectively using an autokerato/refractometer (TONOREF I and II, NIDEK, Gamagori, Japan). To precisely measure the refractive states, automated capture of 3 measurements was repeated at least 4 times, and the mean value was used for analysis. The TONOREF I and II show a specific reliability index of each measurement ranging from 5 to 9, with 9 being the most reliable. When the optic medium is not clear due to corneal edema, anterior chamber inflammation, or cataract, the reliability index decreases. Only measurement values with a high-reliability index of 8 and 9 were included in the analysis. Manifest refractive spherical equivalent was determined as the spherical power plus half the cylindrical power. The magnitude and meridian of the anterior corneal astigmatism were also measured using TONOREF I and II. Furthermore, ocular residual astigmatism which was defined as the difference between manifest refractive astigmatism and anterior corneal astigmatism by Alpins was calculated [24, 25].

$$\mathrm{Ocular residual astigmatism}=\mathrm{manifest refractive astigmatism}-\mathrm{corneal astigmatism}$$

Manifest refractive astigmatism was converted to the value at the corneal plane for calculating ocular residual astigmatism and changed back to the value at the spectacle plane for data presentation. Ocular residual astigmatism was assumed to comprise overall astigmatism mainly due to the toric IOL, as well as IOL tilt/decentration, posterior corneal astigmatism, and other internal optic factors. Ocular residual astigmatism was calculated at the corneal plane. The type of astigmatism (WTR astigmatism, ATR astigmatism, or oblique astigmatism) was also recorded. Corneal astigmatism in which the steeper meridian was between 60° and 120° was defined as WTR astigmatism, that in which the steeper meridian was between 0° and 30° or between 150° and 180° was defined as ATR astigmatism and that in which the steeper meridian was between 30° and 60° or between 120° and 150° was defined as oblique astigmatism. Uncorrected or corrected distance visual acuity on decimal charts was recorded at all visits, and the decimal visual acuity was converted to the logarithm of minimal angle of resolution (logMAR) scale for statistical analysis.

Data on the astigmatism of the manifest refractive, anterior corneal, and ocular residual astigmatism at baseline and at 5 ~ 10 years after baseline were used to analyze the astigmatic change over a period of at least 5 years. The astigmatism was decomposed to vertical (90°)/horizontal (180°) and oblique (45° and 135°) astigmatism components using the X–Y coordinate analysis, originally described by Naeser and Hjortdal [26]. This analysis defines the vertical/horizontal astigmatism component as the Rx and oblique astigmatism components as the Ry. A negative Rx value indicates an ATR astigmatism, while a positive Rx indicates a WTR astigmatism. In the present study, the magnitude and meridian of manifest refractive, anterior corneal, and ocular residual astigmatism at baseline and at 5 ~ 10 years postoperatively were plotted on double-angle plots utilizing the ASCRS Astigmatism Double Angle Plot Tool (available at http://ascrs.org/) [27]. The centroid values of astigmatism at baseline and at 5 ~ 10 years after baseline were also revealed using this tool.

### Statistical analysis

The normality of the data distribution was evaluated by inspecting histograms. The data for the manifest refractive, corneal, ocular residual astigmatism (Rx and Ry values), manifest refractive spherical equivalent, uncorrected and corrected distance logMAR VA, and other continuous variables followed a normal distribution, and therefore, parametric analyses were used in this study. The univariate change in manifest refractive, anterior corneal, and ocular residual astigmatism (Rx and Ry values) between the baseline and 5 ~ 10 years after baseline were compared using a paired *t* test. The bivariate changes in manifest refractive, corneal, and ocular residual astigmatism between the 2 time points were compared using a multivariate analysis of variance (MANOVA) [26]. Statistical power was calculated using the PASS 14 (NCSS LLC, Kaysville, Utah, USA). Differences in the age, preoperative manifest refractive spherical equivalent, preoperative and postoperative uncorrected and corrected distance logMAR VA, and other continuous variables between the ATR and WTR groups were compared using an unpaired *t* test. The ratio of males to females, the ratio of left and right eyes, and other categorical variables between the ATR and WTR groups were compared using the chi-square test or Fisher’s exact test where applicable. Any differences with a *p* value less than 0.05 were considered statistically significant.

## Results

All data of the 120 enrolled eyes were collected. Mean patient age (± standard deviation) was 68.7 ± 8.3 years (range 43 to 84 years), and there were 55 men and 65 women. Preoperative patient characteristics and those at baseline are shown in Table 1. Mean age and the ratio of males to females were significantly higher in the ATR astigmatism group than in the WTR astigmatism group (*p* < 0.0001 and *p* = 0.0032, respectively). Preoperatively, the mean magnitude of the anterior corneal astigmatism and manifest refractive spherical equivalent differed significantly between the ATR and WTR astigmatism groups (*p* ≤ 0.0030). The mean time interval between the surgery and the baseline evaluation and between the baseline evaluation and that at 5 ~ 10 years after baseline did not differ significantly between the ATR and WTR astigmatism groups. At baseline, the magnitude of corneal astigmatism and manifest refractive spherical equivalent did not differ significantly between the 2 groups.

**Table 1:** Comparison of patient characteristics before surgery and at baseline between eyes having against-the-rule (ATR) astigmatism and eyes having with-the-rule astigmatism (WTR)

Characteristics	ATR astigmatism (*n* = 60)	WTR astigmatism (*n* = 60)	*p* value
Preoperatively			
Age	73.12 ± 6.04	64.35 ± 8.09	< 0.0001*
Gender (male/female)	36 M/24F	19 M/41F	0.0032*
Left/right	31L/29R	26L/34R	0.4648
Corneal astigmatism (D)	1.98 ± 0.86	2.48 ± 0.94	0.0030*
MRSE (D) (range)	− 1.23 ± 3.51 (− 16.75–2.13)	− 5.35 ± 5.45 (− 20.25–1.75)	< 0.0001*
LogMAR CDVA	0.41 ± 0.20	0.48 ± 0.31	0.1828
Baseline			
Time interval between surgery and baseline (months)	2.43 ± 0.72	2.53 ± 0.72	0.4502
Time interval between baseline and 5 ~ 10 years after baseline (years)	6.60 ± 1.42	6.83 ± 1.67	0.4107
Corneal astigmatism (D)	1.77 ± 0.81	− 0.76 ± 0.90	0.0605
MRSE (D) (range)	− 0.62 ± 0.66 (− 3.13–0.50)	− 0.76 ± 0.90 (− 5.63–0.63)	0.3356

*M*, male; *F*, female; *D*, diopter; *MRSE*, manifest refractive spherical equivalent; *CDVA*, corrected distance visual acuity; *LogMAR*, logarithm of minimal angle of resolution

^*^Significant difference between eyes having ATR astigmatism and WTR astigmatism

### Univariate and bivariate comparisons of manifest refractive, corneal, and ocular residual astigmatism between the baseline and 5〜10 years after baseline

Univariate comparisons using the paired *t* test indicated that, in the eyes having ATR astigmatism preoperatively, the mean Rx value of manifest refractive astigmatism and corneal astigmatism significantly decreased toward a negative value from the baseline to 5 ~ 10 years after baseline (*p* ≤ 0.0018), and the mean Ry value did not change significantly between the 2 time points (Table 2), indicating an ATR shift of manifest refractive and corneal astigmatism over a period of 5 ~ 10 years. The mean Rx and Ry values of ocular residual astigmatism did not change significantly between the 2 time points. In the eyes with WTR astigmatism, the mean Rx and Ry values of manifest refractive, corneal, and ocular residual astigmatism did not change significantly between the baseline and 5 ~ 10 years after baseline (Table 3).

**Table 2:** Univariate comparison of the mean (± standard deviation) vertical/horizontal (Rx) and oblique (Ry) components of manifest refractive, anterior corneal, and ocular residual astigmatism between the baseline and 5 ~ 10 years after baseline in eyes having against-the-rule (ATR) astigmatism preoperatively

Endpoint	Baseline	5 ~ 10 years after baseline	*p* value
Manifest refractive astigmatism (D)			
Rx (range)	− 0.36 ± 0.34 (− 1.24–0.71)	− 0.59 ± 0.43 (− 1.95–0.12)	0.0018*
Ry (range)	0.04 ± 0.37 (− 1.01–1.36)	0.04 ± 0.38 (− 1.38 − 1.43)	
Anterior corneal astigmatism (D)			
Rx (range)	− 0.76 ± 0.39 (− 1.84–0.34)	− 0.99 ± 0.53 (− 2.76 to − 0.38)	0.0081*
Ry (range)	0.07 ± 0.46 (− 1.92–1.14)	0.09 ± 0.51 (− 2.24–1.41)	0.8953
Ocular residual astigmatism (D)			
Rx (range)	0.44 ± 0.38 (− 0.49–1.31)	0.46 ± 0.48 (− 0.82–2.04)	0.8723
Ry (range)	− 0.04 ± 0.36 (− 1.03–0.91)	− 0.05 ± 0.39 (− 1.36–0.86)	0.8540

*D*, diopters

^*^Statistically significant difference between the 2 time points

**Table 3:** Univariate comparison of the mean (± standard deviation) vertical/horizontal (Rx) and oblique (Ry) components of manifest refractive, anterior corneal, and ocular residual astigmatism between the baseline and 5 ~ 10 years after baseline in eyes having with-the-rule (WTR) astigmatism preoperatively

Endpoint	Baseline	5 ~ 10 years after baseline	*p* value
Manifest refractive astigmatism (D)			
Rx (range)	0.00 ± 0.44 (− 1.36 − 1.23)	0.05 ± 0.48 (− 1.36–1.35)	0.5761
Ry (range)	− 0.10 ± 0.30 (− 0.91 − 0.62)	− 0.07 ± 0.32 (− 0.76–0.65)	0.5539
Anterior corneal astigmatism (D)			
Rx (range)	0.94 ± 0.57 (− 0.17 − 3.00)	0.91 ± 0.55 (− 0.40–2.37)	0.7248
Ry (range)	− 0.12 ± 0.39 (− 1.11–0.81)	− 0.06 ± 0.43 (− 0.82–1.14)	0.4460
Ocular residual astigmatism (D)			
Rx (range)	− 0.95 ± 0.45 (− 2.25–0.27)	− 0.86 ± 0.43 (− 2.05–0.20)	0.2624
Ry (range)	0.03 ± 0.35 (− 0.76–1.12)	0.01 ± 0.49 (− 1.11–1.12)	0.8141

*D*, diopters

**Fig. 1:**
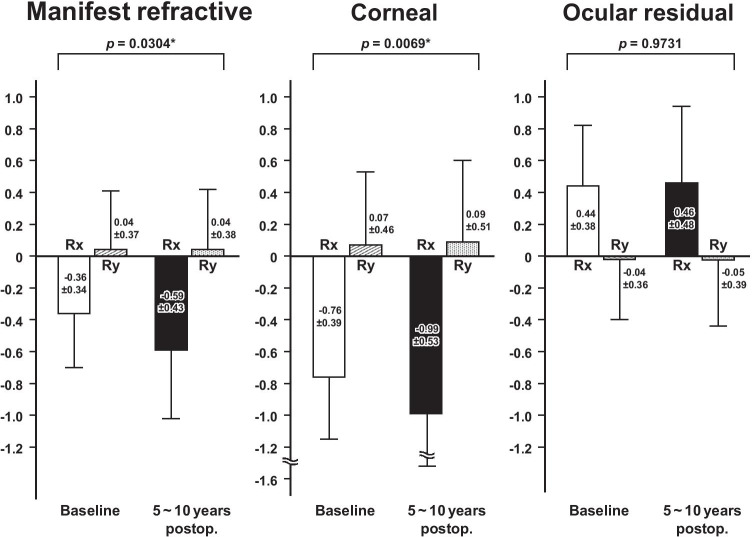
Bivariate comparison of the mean (± standard deviation) vertical/horizontal (Rx) and oblique (Ry) components of the manifest refractive, anterior corneal, and ocular residual astigmatism between the baseline and 5 ~ 10 years after baseline in eyes having against-the-rule astigmatism preoperatively

**Fig. 2:**
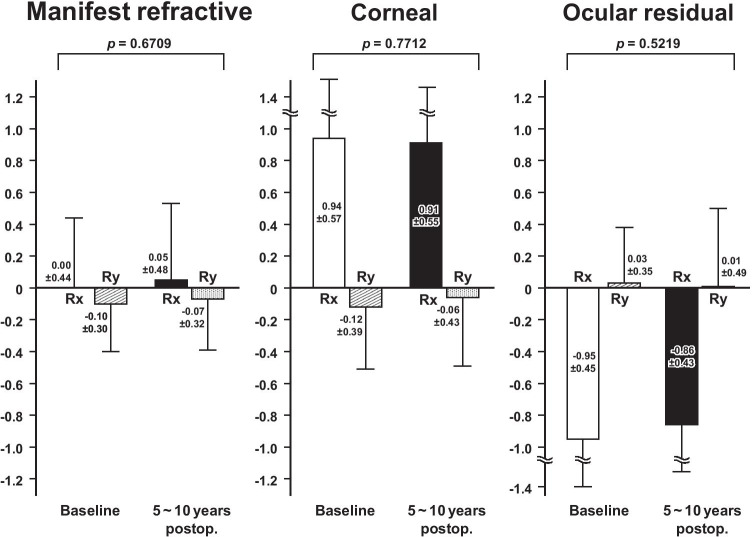
Bivariate comparison of the mean (± standard deviation) vertical/horizontal (Rx) and oblique (Ry) astigmatism components of the manifest refractive, anterior corneal, and ocular residual astigmatism between the baseline and 5 ~ 10 years after baseline in eyes having with-the-rule astigmatism preoperatively

Bivariate comparisons using the MANOVA revealed that, in the eyes having ATR astigmatism preoperatively, the mean Rx and Ry values of manifest refractive astigmatism and anterior corneal astigmatism significantly changed toward ATR astigmatism from baseline to 5 ~ 10 years after baseline (*p* ≤ 0.0304), and the mean Rx and Ry values of ocular residual astigmatism did not change significantly between the 2 time points (Fig. 1). In the eyes having WTR astigmatism preoperatively, the mean Rx and Ry values of manifest refractive, corneal, and ocular residual astigmatism did not change significantly between the 2 time points (Fig. 2). When we assumed an Rx value of 0.20 D and an Ry value of 0.20 D of manifest refractive astigmatism to be a clinically meaningful magnitude of difference between the 2 time points using the MANOVA, power analyses indicated that 60 patients provided a statistical power of 92.4% in eyes having ATR astigmatism and 93.3% in eyes having WTR astigmatism, indicating that the statistical power of the study was sufficient to detect a meaningful change in the main outcome measures.

### Changes in double-angle plots between the baseline and 5 ~ 10 years after baseline

**Fig. 3:**
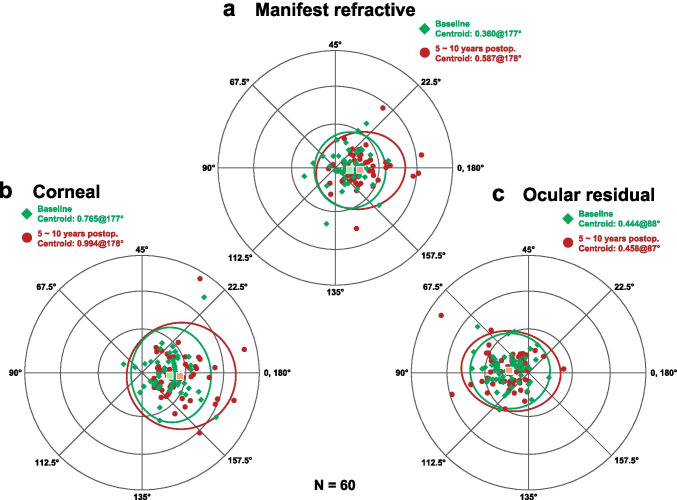
Double-angle plot analysis of the manifest refractive (**a**), anterior corneal (**b**), and ocular residual astigmatism (**c**) between the baseline (green square) and 5 ~ 10 years after baseline (red circle) in eyes having against-the-rule astigmatism preoperatively

**Fig. 4:**
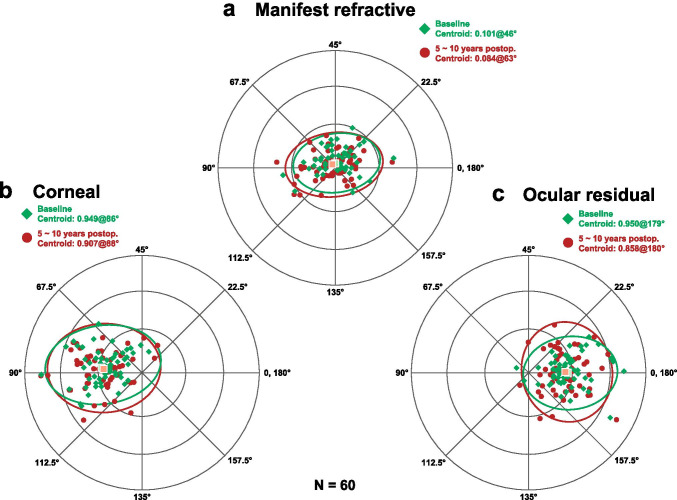
Double-angle plot analysis of the manifest refractive (**a**), anterior corneal (**b**), and ocular residual astigmatism (**c**) between the baseline (green square) and 5 ~ 10 years after baseline (red circle) in eyes having with-the-rule astigmatism preoperatively

The double-angle plots revealed that, in the eyes having ATR astigmatism preoperatively, the manifest refractive astigmatism and anterior corneal astigmatism increased toward an ATR astigmatism from the baseline to 5 ~ 10 years after baseline, and the ocular residual astigmatism did not change markedly (Fig. 3). In the eyes having the WTR astigmatism preoperatively, the manifest refractive, corneal, and ocular residual astigmatism did not change markedly between the 2 time points (Fig. 4).

### Comparison of uncorrected and corrected distance visual acuity between the baseline and 5 ~ 10 years after baseline

In eyes having either ATR and WTR astigmatism preoperatively, the mean uncorrected and corrected distance visual acuity did not worsen from the baseline to 5 ~ 10 years after baseline (Table 4).

**Table 4:** Comparison of the mean (± standard deviation) uncorrected and corrected distance logarithm of minimal angle of resolution (LogMAR) visual acuity between the baseline and the day at 5 ~ 10 years after baseline

Endpoint	Baseline	5 ~ 10 years after baseline	*p* value
Eyes having against-the-rule astigmatism preoperatively			
Uncorrected	0.24 ± 0.23	0.28 ± 0.22	0.3466
Corrected	0.03 ± 0.09	0.04 ± 0.09	0.4489
Eyes having with-the-rule astigmatism preoperatively			
Uncorrected	0.18 ± 0.21	0.24 ± 0.24	0.1486
Corrected	− 0.01 ± 0.07	0.00 ± 0.07	0.7610

## Discussion

The present study revealed that, in the eyes having ATR astigmatism before implantation of a toric IOL, the amount of ATR astigmatism of the manifest refractive astigmatism increased significantly in association with an ATR shift of corneal astigmatism over a period of 5 ~ 10 years after stabilization of the astigmatic correcting effect of a toric IOL. The ocular residual astigmatism, which mainly consists of the effect of a toric IOL, did not differ significantly between the baseline and 5 ~ 10 years after baseline. In the eyes having WTR astigmatism preoperatively, the amounts of manifest refractive, corneal, and ocular residual astigmatism did not worsen over the 5 ~ 10 years after baseline. These findings suggest that the long-term effects of a toric IOL for correcting the manifest refractive astigmatism deteriorate due to the ATR shift of corneal astigmatism in eyes having ATR astigmatism, while they are maintained, at least over 5 ~ 10 years postoperatively, in the eyes having WTR astigmatism.

Mean uncorrected distance visual acuity did not worsen between the baseline and 5 ~ 10 years after baseline in eyes having either ATR or WTR astigmatism. This finding indicates that the change in manifest refractive astigmatism over 5 ~ 10 years postbaseline did not significantly affect uncorrected visual acuity. The mean follow-up duration, however, was 6.6 years in eyes having ATR astigmatism and 6.8 years in eyes having WTR astigmatism. Because an ATR shift in corneal and refractive astigmatism progresses with age [28–32], uncorrected distance visual acuity must deteriorate beyond 10 years. Furthermore, the mean change in manifest refractive astigmatism was 0.23D in eyes having ATR astigmatism and 0.05D in eyes having WTR astigmatism. Thus, because the difference in the astigmatic change between eyes having ATR and WTR astigmatism was small, it did not lead to a marked difference in impairment of uncorrected visual acuity.

Corneal astigmatism is mainly WTR astigmatism in younger individuals and continues to change toward ATR astigmatism with advancing age [28–31, 33]. On average, the type of corneal astigmatism changes from WTR astigmatism to ATR astigmatism at 60 years of age [28–31]. This age-related ATR change in corneal astigmatism also occurs to a similar extent in eyes that undergo cataract surgery [28, 29]. In the present study, because the mean age of eyes having ATR astigmatism before surgery was 73.1 ± 6.0 years, the type of corneal astigmatism had already changed to ATR astigmatism at the time of surgery. Accordingly, the increase in ATR corneal astigmatism directly led to an increase in manifest refractive astigmatism. In contrast, because the mean age of eyes having WTR astigmatism was 64.4 ± 8.1 years, the type of corneal astigmatism had not yet shifted from WTR astigmatism to ATR astigmatism in most eyes. Thus, because the type of corneal astigmatism was shifting during the study period, the change in manifest refractive astigmatism might not be apparent in eyes having WTR astigmatism. Furthermore, ocular residual astigmatism, which mainly consists of the astigmatism-correcting effect of the toric IOL, did not change significantly over 5 ~ 10 years postoperatively. Because corneal astigmatism continues to change toward ATR astigmatism over 20 years [30], however, the effect of a toric IOL will deteriorate over time, even in eyes with WTR astigmatism.

Previous studies showed that the astigmatism-correcting effect and rotational stability of toric IOLs are well maintained during a 1- or 2-year follow-up time period [14–18]. For example, Miyake et al. [16] reported excellent visual outcomes and rotational stability for up to 2 years postoperatively. No studies to date, however, have evaluated long-term changes in the visual outcomes and refractive effects of toric IOLs for 5 or more years after stabilization of surgically induced astigmatic changes. The present study revealed that the effects of the toric IOL for correcting manifest refractive astigmatism significantly deteriorated with age due to the ATR change in corneal astigmatism, specifically in eyes with ATR astigmatism before surgery.

The present study had several limitations. First, because this study was conducted in a retrospective manner, some of the patient characteristics, including age and sex, differed significantly between the ATR and WTR groups. Corneal astigmatism changes from WTR astigmatism to ATR astigmatism with age [28–31, 33], and this ATR change occurs faster in men than in women [31]; therefore, the distribution of WTR and ATR astigmatism basically differs depending on age and sex. Accordingly, it was difficult to match the age and sex of eyes that underwent implantation of a toric IOL between the ATR and WTR astigmatism groups. Second, because the follow-up duration was only 5 ~ 10 years, no significant change in uncorrected and corrected distance visual acuity was detected. Changes in the visual outcomes after toric IOL implantation should be examined over a longer time period. Third, we did not examine the long-term refractive changes in eyes having oblique astigmatism. Because only a small number of eyes had the necessary degree of oblique astigmatism for toric IOL implantation during the study period, we did not implant a toric IOL in enough eyes having oblique astigmatism for analysis. Further studies are necessary to evaluate the long-term refractive effect of a toric IOL in eyes having oblique astigmatism.

In conclusion, the refractive effect of the toric IOL significantly deteriorated over time in association with an ATR change of corneal astigmatism in eyes with ATR astigmatism, while it was maintained in eyes with WTR astigmatism. Because the effect of a toric IOL deteriorates with age, overcorrection of ATR astigmatism should be considered depending on the age and sex of the patients scheduled for toric IOL implantation. Further studies are needed to investigate the effects of the toric IOL on visual outcomes for at least 10 years postoperatively.

## Supplementary Information

Below is the link to the electronic supplementary material.

Supplementary file1 (XLSX 54 kb)

## Data Availability

All data that support the findings of this study are available on request from the corresponding author.
